# Technology and Tool Development for BACPAC: Qualitative and Quantitative Analysis of Accelerated Lumbar Spine MRI with Deep-Learning Based Image Reconstruction at 3T

**DOI:** 10.1093/pm/pnad035

**Published:** 2023-03-21

**Authors:** Misung Han, Emma Bahroos, Madeline E Hess, Cynthia T Chin, Kenneth T Gao, David D Shin, Javier E Villanueva-Meyer, Thomas M Link, Valentina Pedoia, Sharmila Majumdar

**Affiliations:** Department of Radiology and Biomedical Imaging, University of California, San Francisco, San Francisco, CA, United States; Department of Radiology and Biomedical Imaging, University of California, San Francisco, San Francisco, CA, United States; Department of Radiology and Biomedical Imaging, University of California, San Francisco, San Francisco, CA, United States; Department of Radiology and Biomedical Imaging, University of California, San Francisco, San Francisco, CA, United States; Department of Radiology and Biomedical Imaging, University of California, San Francisco, San Francisco, CA, United States; UC Berkeley-UCSF Graduate Program in Bioengineering, University of California, San Francisco, CA, United States; Applications and Workflow, GE Healthcare, Menlo Park, CA, United States; Department of Radiology and Biomedical Imaging, University of California, San Francisco, San Francisco, CA, United States; Department of Radiology and Biomedical Imaging, University of California, San Francisco, San Francisco, CA, United States; Department of Radiology and Biomedical Imaging, University of California, San Francisco, San Francisco, CA, United States; UC Berkeley-UCSF Graduate Program in Bioengineering, University of California, San Francisco, CA, United States; Department of Radiology and Biomedical Imaging, University of California, San Francisco, San Francisco, CA, United States; UC Berkeley-UCSF Graduate Program in Bioengineering, University of California, San Francisco, CA, United States

**Keywords:** lumbar spine MRI, lower back pain, fast acquisition, deep learning reconstruction, clinical MRI, segmentation

## Abstract

**Objectives:**

To evaluate whether combining fast acquisitions with deep-learning reconstruction can provide diagnostically useful images and quantitative assessment comparable to standard-of-care acquisitions for lumbar spine magnetic resonance imaging (MRI).

**Methods:**

Eighteen patients were imaged with both standard protocol and fast protocol using reduced signal averages, each protocol including sagittal fat-suppressed T_2_-weighted, sagittal T_1_-weighted, and axial T_2_-weighted 2D fast spin-echo sequences. Fast-acquisition data was additionally reconstructed using vendor-supplied deep-learning reconstruction with three different noise reduction factors. For qualitative analysis, standard images as well as fast images with and without deep-learning reconstruction were graded by three radiologists on five different categories. For quantitative analysis, convolutional neural networks were applied to sagittal T_1_-weighted images to segment intervertebral discs and vertebral bodies, and disc heights and vertebral body volumes were derived.

**Results:**

Based on noninferiority testing on qualitative scores, fast images without deep-learning reconstruction were inferior to standard images for most categories. However, deep-learning reconstruction improved the average scores, and noninferiority was observed over 24 out of 45 comparisons (all with sagittal T_2_-weighted images while 4/5 comparisons with sagittal T_1_-weighted and axial T_2_-weighted images). Interobserver variability increased with 50 and 75% noise reduction factors. Deep-learning reconstructed fast images with 50% and 75% noise reduction factors had comparable disc heights and vertebral body volumes to standard images (*r*^2^≥ 0.86 for disc heights and *r*^2^≥ 0.98 for vertebral body volumes).

**Conclusions:**

This study demonstrated that deep-learning-reconstructed fast-acquisition images have the potential to provide noninferior image quality and comparable quantitative assessment to standard clinical images.

## Introduction

Lower back pain (LBP) is a major cause of disability responsible for limiting work related activities and ultimately a cause of deteriorating quality of life.^1^ The impact of LBP results in loss of work productivity and the healthcare expenditure alone ranges from $50 to $91.8 billion yearly in the United States.^2^^,^^3^ Magnetic resonance imaging (MRI) is extensively used in the clinical evaluation of LBP, diagnosing abnormalities in the vertebral bodies, intervertebral discs, nerve roots, and spinal cord related to degenerative disease, osteoporotic compression fracture, trauma, infectious and inflammatory conditions, and tumors. Even though imaging findings of abnormalities are not always associated with LBP,^4–6^ a growing number of studies have demonstrated that MRI findings can function as important biomarkers associated with LBP.^7–9^

Currently, a standard clinical lumbar spine MRI protocol consists of high-resolution T_1_-weighted and T_2_-weighted 2D fast-spin echo (FSE) sequences in multiple orientations and requires approximately 30-minute scan time. When intravenous contrast injection is required, additional post-contrast sequences are acquired, further increasing the total scan time. Remaining still in the MRI scanner for a long duration can be a challenge for patients particularly when in pain, and can generate motion artifacts. Reducing acquisition time would improve patient experience and image quality in the setting of LBP. Furthermore, a shorter clinical protocol time would allow for adding special quantitative imaging^10^^,^^11^ as part of routine imaging to look beyond structural abnormalities.

Numerous strategies have been developed for fast acquisition; partial Fourier,^12^^,^^13^ parallel imaging,^14^^,^^15^ and compressed sensing,^16^ which exploit *k*-space data redundancy or spatial correlation, are the most common schemes to shorten acquisition times. However, these acceleration approaches suffer from reduced signal-to-noise ratio (SNR) or blurring,^17–19^ and application of those would not provide sufficient imaging quality for clinical 2D FSE lumbar spine imaging that normally uses multiple signal averaging (number of excitations [NEX] > 1) to achieve sufficient SNR.

Recently, deep-learning (DL) based methods have been rapidly developed for various MRI research areas including undersampled data reconstruction,^20^^,^^21^ segmentation,^22^ super-resolution,^23^ and denoising.^24^ In particular, a DL reconstruction method employing a deep convolutional neural network, which directly applies to raw *k*-space data, has been developed to improve SNR and to reduce ringing artifacts (commercially available as AIR Recon DL, GE Healthcare).^25^ The feasibility of this particular DL reconstruction to improve SNR while providing similar or improved diagnostic capability compared to conventional reconstruction has been validated for high-resolution post-operative pituitary imaging,^26^ late gadolinium enhancement cardiac imaging,^27^ and clinical assessment of the prostate cancer,^28^ the peripheral nerve,^29^ and the hip and shoulder,^30^^,^^31^ but has not been validated comprehensively for clinical lumbar spine MRI at 3 T yet.

In this study we evaluated the application of DL reconstruction to fast, reduced-NEX acquisitions for 2D clinical lumbar spine MRI in comparison to standard acquisitions through both qualitative and quantitative analysis. Qualitative analysis was based on radiologists’ evaluation. Quantitative analysis was performed by applying previously developed DL-based segmentation algorithms to segment intervertebral discs and vertebral bodies and to compare extracted disc heights and vertebral body volumes,^32^ which can be important biomarkers associated with spine degeneration^33–35^ and biomechanical modeling.^36^

## Materials and methods

### Image acquisition and reconstruction

This study was conducted in accordance with and approval by the local institution review board with waived informed consent. Images and raw *k*-space data from 18 patients with LBP, scanned between March to June 2020 at our institution, were retrospectively collected. A GE 3 T SIGNA Premier MRI scanner and a table-embedded 32-channel spine posterior coil array (GE Healthcare, Waukesha, WI, USA) were used for imaging.

Two different clinical protocols were used at our institution for lumbar spine MRI, a musculoskeletal (MSK) radiology lumbar spine protocol and a neuroradiology lumbar spine protocol. The MSK radiology protocol was tailored for better depiction of orthopedic and rheumatologic disorders while the neuroradiology protocol for better depiction of spinal cord and peripheral nerve disorders (exploits a longer TE for T_2_-weighted sequences). These standard lumbar spine protocols consisted of sagittal fat-saturated T_2_-weighted 2D fast spin-echo (FSE) (SAG T2 FS) sequence, sagittal T_1_-weighted 2D FSE (SAG T1) sequence, axial T_2_-weighted 2D FSE (AX T2) sequence, axial T_1_-weighted 2D FSE sequence, and coronal T_1_-weighted 2D FSE sequence. These clinical sequences used a NEX of 1–2 to achieve SNR for a high diagnostic confidence, and all imaging parameters were within ranges recommended by the Back Pain Consortium (BACPAC) Spine Imaging Working Group.^37^ For each patient, imaging using one of these clinical protocols based on physicians’ order was performed, and then imaging with a fast protocol consisting of SAG T2 FS, SAG T1, and AX T2 sequences, with only a NEX reduced to 0.5–1, was also performed. With 0.5 NEX prescription, partial Fourier was applied along the *k*_y_ dimension.^12^^,^^13^Table 1 shows image parameters for SAG T2 FS, SAG T1, and AX T2 sequences for both MSK radiology and neuroradiology protocols, with denoting NEX for both standard and fast acquisition protocols and approximate scan times.

**Table 1.** MSK radiology and neuroradiology lumbar spine protocols

	MSK Radiology Protocol			Neuroradiology Protocol		
Sequences	SAG T2 FS	SAG T1	AX T2	SAG T2 FS	SAG T1	AX T2
TE [ms]	67	8	60	78	8	96
TR [ms]^a^	5000	700	6000	5000	700	6000
FOV [cm]	26	26	18	26	26	18
Slice thickness [mm]	3	3	4	3	3	4
Slice number	20–32	20–32	45–84	20–32	20–32	45–84
Echo train length	16	4	16	16	4	18
Matrix size	392 × 224	352 × 224	256 × 192	384 × 224	352 × 192	292 × 192
Readout b bandwidth [kHz]	±83.3	±83.3	±83.3	±100	±100	±100
NEX	2 **(1)**	2 **(0.5)**	1 **(0.5)**	2 **(1)**	2 **(0.5)**	1 **(0.5)**
Scan time [mins: secs]^b^	4:30 **(2:15)**	3:30 **(1:20)**	4:30 **(2:30)**	4:30 **(2:15)**	3:30 **(1:20)**	4:30 **(2:30)**

a Depending on patient weights, the TR and resultant scan times slightly varied, but the average values are indicated.

b The NEX and scan time for standard clinical and fast protocols are denoted outside and within parentheses, respectively.

The vendor-supplied prototype version of AIR Recon DL was applied to raw *k*-space data from the reduced-NEX fast acquisition protocol offline. AIR Recon DL pipeline included a deep convolutional neural network with 4.4 million parameters in approximately 10 000 kernels, and designed to improve input data’s SNR while enhancing sharpness and reducing ringing artifacts.^25^ The deep convolution neural network was trained using pairs of images representing near-perfect images (high-resolution images with minimal ringing and very low noise levels) and synthesized images of lower resolution versions with more truncation artifacts and higher noise levels.^25^ Data with 0.5 NEX acquisition was also included for training data sets. Image augmentation included rotations and flips, intensity gradients, phase manipulations, and additional Gaussian noise, to add robustness, and over 4 million unique image/augmentation combinations over various anatomy were used for training.^25^ The raw data was reconstructed using the algorithm with three different noise reduction factors, 25%, 50%, and 75%. Vendor's standard reconstruction computer with multiple CPU cores was used for reconstruction, and each DL reconstruction took approximately one minute.

### Qualitative assessment and statistical analysis

For all of three sequences (SAG T2 FS, SAG T1, and AX T2), original images from the standard clinical acquisitions and fast acquisitions, and DL-reconstructed images from the fast acquisitions with 25%, 50%, and 75% noise reduction factors (will name those as Standard images, Fast images, and Fast-DL images (or Fast DL25, Fast DL50, and Fast DL75 images), respectively) were evaluated. Patient information was anonymized, and image details such as the NEX, the application of DL reconstruction algorithm, and DL reconstruction noise reduction factor, were all removed from the DICOM header. Qualitative image assessment was performed by three board-certified radiologists specializing in neuroradiology or MSK radiology, CC (neuroradiologist), TML (MSK radiologist) and JVM (neuroradiologist), with 25, 26, and 5 years of experience, respectively.

All image series from the two lumbar spine protocols were randomly ordered for each sequence (90 series for each sequence, five series for each of 18 patients) and presented to the radiologists. The radiologists graded each series sequentially for five different categories, “Apparent SNR,” “Ability to Discern Anatomical Structures,” “Diagnostic Confidence,” “Overall Image Quality,” and “Presence of Artifacts.” The first four categories were rated on a Likert scale of 1 to 5 (1–5: poor to excellent), while the last metric was rated as binary, 0 (no artifacts) and 1 (artifacts), as described in Table 2. Radiologists were blinded to each other’s rating.

**Table 2.** Ratings for apparent SNR, ability to discern anatomical structure, diagnostic confidence, overall image quality, and presence of artifacts, used for radiologists’ qualitative assessment

Score	Apparent SNR^a^	Ability to Discern Anatomical Structure^a^	Diagnostic Confidence^a^	Overall Image Quality^a^	Presence of Artifacts^b^
0	-	-	-	-	No
1	Too noisy, image uninterpretable	Not visible	No diagnostic value	Terrible	Yes
2	Noise moderately affects interpretation	Barely visible	Very limited diagnostic value	Poor	-
3	Noise mildly limits interpretation	Adequately visible	Acceptable for most diagnosis	Average	-
4	No adverse effects on interpretation	Good visibility	Good for majority of diagnosis	Good	-
5	Optimal SNR	Excellent visibility	Optimal	Excellent	-

a Rated on a Likert scale of 1 to 5.

b Rated as binary (0 or 1).

Noninferiority testing^38^^,^^39^ was performed to compare the average scores of Fast and Fast-DL images from the three radiologists to those from Standard images. The null and alternative hypotheses for the first four categories were: H_0_: $\mu_{D}-\mu_{S}\leq -\delta_{l}$ and H_1_: $\mu_{D}-\mu_{S}>-\delta_{l}$ where $\mu_{D}\;$represents the average scores from Fast or Fast-DL groups while $\mu_{S}\;$represents those from Standard groups. The noninferiority margin $-\delta_{l}$ was set as -0.5. For the “Presence of Artifacts,” the null and alternative hypotheses were: H_0_: $\mu_{D}-\mu_{S}\geq \delta_{b}$ and H_1_: $\mu_{D}-\mu_{S}<\delta_{b}$ where the noninferiority margin $\delta_{b}$ was set as 0.2 (as a lower difference was improvement with this category). A one-sided Wilcoxon signed-rank test was performed to assess noninferiority, and *P* values were calculated using normal approximation and continuity correction.^40^^,^^41^ The *P* values were adjusted using the false discovery rate (FDR) to correct for multiple testing.^42^

Interobserver agreement of scores by the three radiologists for each category and each sequence was conducted using a Conger’s kappa (κ) coefficient with quadratic weighting.^43^ The coefficient was interpreted as follows: κ < 0, no agreement; 0 < κ ≤ 0.2, slight agreement; 0.2 < κ ≤ 0.4, fair agreement; 0.4 < κ ≤ 0.6, moderate agreement; 0.6 < κ ≤ 0.8, substantial agreement; and 0.8 < κ ≤ 1, almost perfect agreement.

### Automatic segmentation and quantitative analysis

Two convolutional neural networks incorporating 2D V-Net architecture,^44^ previously trained with 27 and 24 SAG T1 patient data sets (for intervertebral discs and vertebral bodies respectively)^32^ were used to segment intervertebral discs and vertebral bodies separately. After acquiring segmentation masks on each image slice, 2D slice masks were reconstructed into 3D volume masks and postprocessed to smooth, fill holes, and identify largest connected components. Disc segmentation masks were used to calculate intervertebral disc height by computing a 3D centroid for each disc to find the most central slice and extracting the shortest side length from a minimum bounding rectangle of the disc segmentation. The vertebral body segmentation masks were used to calculate each vertebral body volume by summing the number of foreground pixels for each body and converting to patient-based space. Segmented discs (mostly located between T12 and S1 vertebral bodies) or vertebral bodies (among T12-S1 vertebral bodies) were manually matched between Standard, Fast, and Fast-DL images. These derived quantities from Fast and Fast-DL images were compared to those from Standard images using correlation analysis.

## Results

### Patient population

The study included 18 patients (10 males and 8 females), with patient age ranging from 31 to 82 years (median age of 61 years). Thirteen out of 18 patients were imaged with the MSK radiology protocol while the other five patients were imaged with the neuroradiology protocol. Various common degenerative spine abnormalities were demonstrated on their MR images such as Modic changes in vertebral endplates, disc osteophytes, annular fissures, disc protrusion, disc extrusion, spinal and foraminal stenoses, and facet arthropathy and cyst, and some patients had previous surgery or metal implants.

### Reconstructed images with and without DL reconstruction

**Figure 1.**
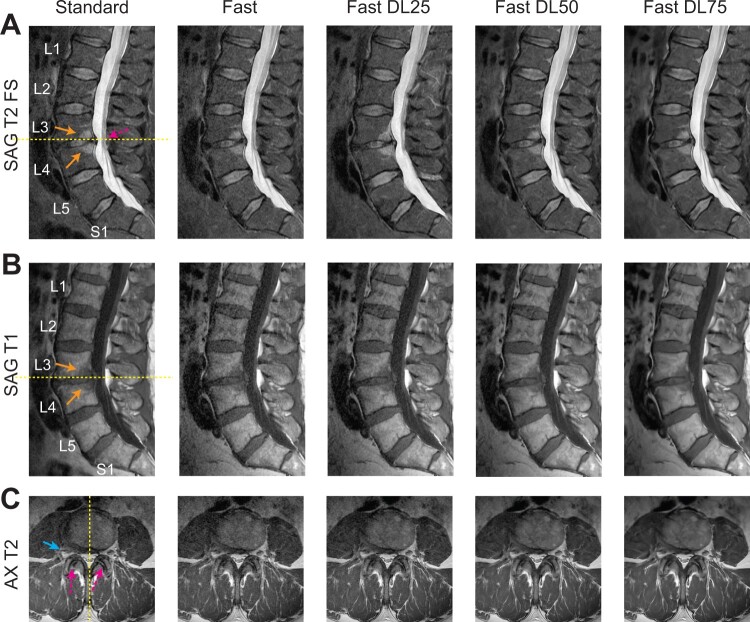
Comparison of images from a 63-year old male patient acquired with the MSK radiology lumbar spine protocol. Standard, Fast, Fast DL25, Fast DL50, Fast DL75 images from SAG T2 FS (**A**), SAG T1 (**B**), and AX T2 (**C**) are all shown with the axial and sagittal locations denoted by dashed lines in the left column. In (**A**, **B**) the Modic type I changes and mild central canal stenosis are denoted (by solid arrows and dashed arrows, respectively). In (**C**), mild facet arthropathy and moderate right foraminal narrowing are denoted by dashed arrows and a solid arrow, respectively.

Figure 1 compares Standard, Fast, and Fast-DL images from a 63-year-old male patient with LBP for more than six weeks, acquired with the MSK lumbar spine protocol. Sagittal images (Figure 1A–B) show Modic type I endplate changes in the posterior L3 and L4 vertebral bodies (fibrovascular endplate change) and an L3–L4 disc extrusion resulting in mild central canal stenosis. On axial images at L3–L4 disc (Figure 1C), mild facet arthropathy and moderate right foraminal narrowing were identified. Fast images provided a lower SNR than Standard images as expected, but the reduction of noise was perceived with Fast-DL images while degeneration in vertebral bodies, discs, spinal cord, and facet joints was well visualized.

**Figure 2.**
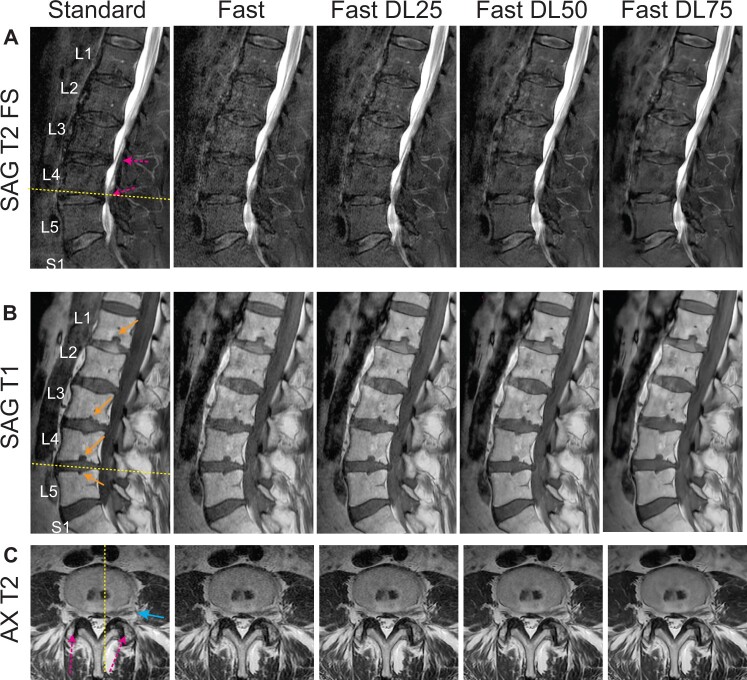
Comparison of images from a 73-year old male patient acquired with the neuroradiology lumbar spine protocol. Standard, Fast, Fast DL25, Fast DL50, Fast DL75 images from SAG T2 FS (**A**), SAG T1 (**B**), and AX T2 (**C**) are again shown. Mild central canal stenosis and small Schmorl’s nodes are denoted by dashed arrows (**A**) and solid arrows (**B**). In the axial images (**C**), mild facet arthropathy and mild left foraminal narrowing related to a lateral disc protrusion are identified (denoted by arrows).

Images from a 73-year-old male patient acquired with the neuroradiology protocol are shown in Figure 2. SAG T2 FS images show decreased contrast over discs and intervertebral bodies while increased contrast between spinal cord and cerebrospinal fluid compared to Figure 1 due to a higher TE. This patient had dessiccated (dehydrated) intervertebral discs and small Schmorl's nodes (endplate disc herniations) in the L1, L3, L4, and L5 vertebral bodies (Figure 2B). Disc protrusions and mild-moderate central canal stenoses at L3–L4 and L4–L5 discs (Figure 2A) and mild facet arthropathy with mild left foraminal narrowing (Figure 2C) were also identified. These abnormalities were again well-visualized on Fast-DL images.

**Figure 3.**
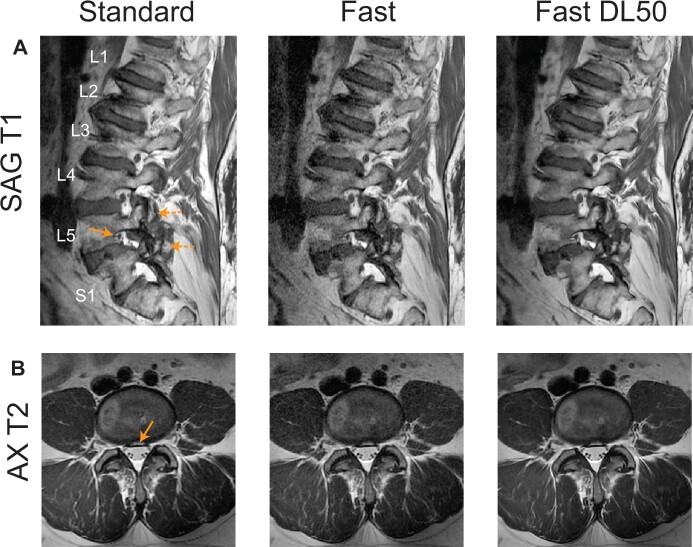
Comparison of other anatomical structures/pathologies. (**A**) Images from a patient having transitional lumbosacral anatomy, moderate neuroforaminal stenosis at the L5–S1 disc level (solid arrow), and severe L4 and L5 facet arthropathy (dashed arrows). (B) Images from a patient having central annular fissure (solid arrow). These abnormal structures were well delineated on Fast DL50 images.

Figure 3 compares Standard, Fast, and Fast DL50 images from other two patients (78 year-old and 39 year-old males) visualizing different anatomical structures/pathologies. The patient in Figure 3A had transitional lumbosacral anatomy and had moderate neuroforaminal stenosis at the L5–S1 disc level and severe L4 and L5 facet arthropathy. Figure 3B shows central annular fissure (bright signal depicted by an arrow). These abnormal structures were well delineated with Fast DL50 images again.

### Qualitative assessment

**Figure 4.**
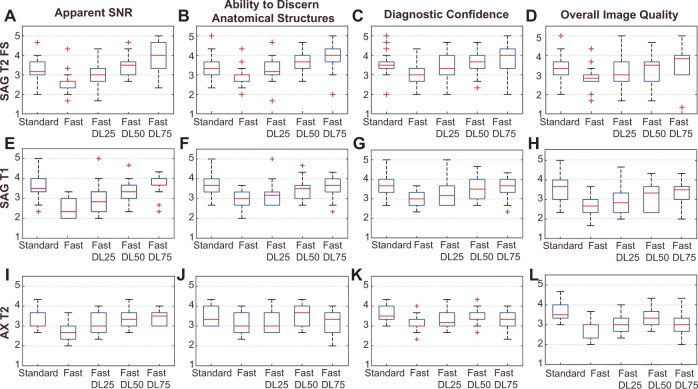
Box plots summarizing qualitative assessment. Radiologists’ scores over the 18 patients for the Standard, Fast, Fast DL25, Fast DL50, and Fast DL75 groups are compared as box plots on “Apparent SNR,” “Ability to Discern Anatomical Structures,” “Diagnostic confidence,” and “Overall Image Quality,” separately for SAG T2 FS (**A–D**), SAG T1 (**E–H**), and AX T2 (**I–L**) sequences. The minimum, first quartile, median, third quartile, and maximum values are shown, and outliers are denoted as red crosses.

The mean Likert scores over the three radiologists for all 18 patients are summarized in Figure 4 as box plots for the categories of “Apparent SNR,” “Ability to Discern Anatomical Structures,” “Diagnostic Confidence,” and “Overall Image Quality.” The means and standard deviations of the average scores from the three radiologists are presented in Table 3 for the five categories including the “Presence of Artifacts.” The mean scores from the Fast groups were lower than those from the Standard groups for the first four categories, but with DL reconstruction, the mean scores improved.

**Table 3.** Summary of the mean scores over the three radiologists

	Apparent SNR	Anatomical Structures	Diagnostic Confidence	Overall Image Quality	Presence of Artifacts
SAG T2 FS					
Standard	3.30 ± 0.61	3.50 ± 0.63	3.56 ± 0.68	3.37 ± 0.67	0.54 ± 0.30
Fast	2.61 ± 0.62	2.91 ± 0.56	3.06 ± 0.61	2.81 ± 0.64	0.50 ± 0.24
Fast DL25	3.10 ± 0.66	3.26 ± 0.66	3.35 ± 0.60	3.19 ± 0.75	0.49 ± 0.27
Fast DL50	3.52 ± 0.60	3.60 ± 0.58	3.61 ± 0.63	3.35 ± 0.80	0.65 ± 0.21
Fast DL75	3.94 ± 0.73	3.85 ± 0.65	3.87 ± 0.69	3.63 ± 0.83	0.67 ± 0.28
SAG T1					
Standard	3.63 ± 0.75	3.76 ± 0.61	3.72 ± 0.59	3.59 ± 0.74	0.57 ± 0.30
Fast	2.52 ± 0.49	2.96 ± 0.44	2.96 ± 0.39	2.63 ± 0.53	0.76 ± 0.28
Fast DL25	2.98 ± 0.76	3.22 ± 0.60	3.28 ± 0.64	2.94 ± 0.69	0.74 ± 0.27
Fast DL50	3.31 ± 0.62	3.46 ± 0.58	3.46 ± 0.61	3.22 ± 0.71	0.80 ± 0.26
Fast DL75	3.63 ± 0.50	3.54 ± 0.56	3.54 ± 0.56	3.31 ± 0.65	0.76 ± 0.30
AX T2					
Standard	3.30 ± 0.57	3.52 ± 0.45	3.63 ± 0.39	3.61 ± 0.47	0.57 ± 0.22
Fast	2.74 ± 0.51	3.13 ± 0.49	3.17 ± 0.45	2.85 ± 0.50	0.63 ± 0.25
Fast DL25	3.13 ± 0.49	3.20 ± 0.53	3.31 ± 0.49	3.09 ± 0.48	0.76 ± 0.22
Fast DL50	3.41 ± 0.49	3.44 ± 0.57	3.48 ± 0.42	3.30 ± 0.40	0.81 ± 0.23
Fast DL75	3.46 ± 0.36	3.15 ± 0.57	3.30 ± 0.48	3.06 ± 0.61	0.85 ± 0.21

The mean scores and standard deviations over the 18 patients are presented for the Standard, Fast, Fast DL25, Fast DL50, and Fast DL75 groups on five different categories.

Table 4 shows the results of the noninferiority tests. The (Pseudo)median^40^ of the difference scores between Fast/Fast DL groups and Standard groups as well as the *P* values from the one-sided tests for the specified noninferior margins are indicated. The mean scores from Fast groups were statistically inferior to those from standard groups for four to five categories of all sequences. However, with Sag T2 FS, all Fast DL groups with the three different noise reduction factors showed statistical noninferiority compared to the Standard groups across all categories. For SAG T1, Fast DL25 and Fast DL50 images received inferior scores for most of categories, while Fast DL75 images showed noninferiority for “Apparent SNR,” “Ability to Discern Anatomical Structures,” and “Diagnostic Confidence.” For AX T2, Fast DL50 images attained statistically noninferior scores for “Apparent SNR,” “Ability to Discern Anatomical Structures,” and “Diagnostic Confidence.”

**Table 4.** Noninferiority tests comparing scores of Fast and Fast-DL groups with those of Standard group on five different categories

	Apparent SNR		Anatomical Structures		Diagnostic Confidence		Overall Image Quality		Presence of Artifacts	
	(Pseudo) median^a^	*P* value^b^	(Pseudo) median^a^	*P* value^b^	(Pseudo) median^a^	*P* value^b^	(Pseudo) median^a^	*P* value^b^	(Pseudo) median^a^	*P* value^b^
SAG T2 FS										
Fast vs St.	−0.667	.894	−0.500	.788	−0.500	.456	−0.500	.587	0.000	.002*
Fast DL25 vs St.	−0.167	.013*	−0.167	.001*	−0.167	.001*	−0.167	.007*	0.000	.003*
Fast DL50 vs St.	0.167	.001*	0.167	.001*	0.000	.000*	0.000	.013*	0.000	.007*
Fast DL75 vs St.	0.667	.000*	0.333	.001*	0.333	.000*	0.333	.002*	0.000	.006*
SAG T1										
Fast vs St.	−1.167	1.000	−0.833	.986	−0.833	.972	−1.000	.999	0.167	.123
Fast DL25 vs St.	−0.667	1.000	−0.667	.893	−0.500	.562	−0.667	.999	0.167	.123
Fast DL50 vs St.	−0.333	.079	−0.333	.087	−0.333	.046*	−0.333	.210	0.167	.268
Fast DL75 vs St.	0.000	.019*	−0.167	.033*	−0.167	.029*	−0.333	.201	0.167	.123
AX T2										
Fast vs St.	−0.500	.703	−0.333	.109	−0.500	.319	−0.667	.984	0.000	.010*
Fast DL25 vs. St.	−0.167	.006*	−0.333	.129	−0.333	.195	−0.500	.914	0.167	.430
Fast DL50 vs St.	0.167	.001*	0.000	.014*	−0.167	.014*	−0.333	.320	0.333	.716
Fast DL75 vs St.	0.167	.001*	−0.333	.129	−0.333	.195	−0.500	.914	0.333	.716

a (Pseudo)median of difference scores between Fast/Fast-DL groups and Standard groups.

b *P* values of noninferior tests (one-sided Wilcoxon singed-rank tests) with an inferior bound of −0.5 for the first four criteria and 0.25 for “Presence of Artifacts.”

* Indicates statistical noninferiority (*P *<* *.05) based on noninferior tests.

Conger’s κ coefficients to check interobserver agreement are shown in Table 5. There was no to slight agreement on “Presence of Artifacts” (κ < 0.2), but for other criteria, fair to moderate agreement was demonstrated for SAG T2 (0.255 < κ < 0.550), and slight to moderate agreement for SAG T1 (0.051 < κ < 0.550). The AX T2 sequence exhibited less agreement (−0.003 < κ < 0.516) than the other two sequences. Overall, Fast DL75 groups and Fast DL50 groups showed the least agreement among the radiologists, suggesting varying levels of perception of DL-reconstructed images.

**Table 5.** Conger’s κ coefficients to assess interobserver variability

	Apparent SNR	Anatomical Structures	Diagnostic Confidence	Overall Image Quality	Presence of Artifacts
SAG T2 FS					
Standard	**0.427^a^**	0.379	**0.512**	**0.549**	0.176
Fast	**0.517**	0.378	0.348	**0.435**	0.071
Fast DL25	**0.419**	**0.488**	**0.481**	**0.531**	0.087
Fast DL50	0.234	0.371	**0.421**	**0.506**	0.007
Fast DL75	0.255	0.336	**0.413**	0.331	0.126
SAG T1					
Standard	**0.515**	**0.488**	**0.449**	**0.479**	0.140
Fast	0.300	0.255	0.158	0.300	0.156
Fast DL25	**0.552**	**0.482**	**0.447**	**0.458**	0.118
Fast DL50	0.222	0.369	0.377	0.397	0.143
Fast DL75	0.052	0.216	0.216	0.192	0.250
AX T2					
Standard	0.266	0.381	0.162	0.292	0.046
Fast	0.376	**0.516**	0.319	0.353	0.046
Fast DL25	0.184	0.327	0.239	0.229	−0.031
Fast DL50	0.078	0.219	0.121	0.073	0.122
Fast DL75	−0.003	0.166	0.069	0.124	0.045

The coefficients were interpreted as follows: κ < 0, no agreement; 0 < κ ≤ 0.2, slight agreement; 0.2 < κ ≤ 0.4, fair agreement; 0.4 < κ ≤ 0.6, moderate agreement; 0.6 < κ ≤ 0.8, substantial agreement; and 0.8 < κ ≤ 1, almost perfect agreement.

a Bold text was used with agreement > 0.4.

### Automatic segmentation and quantitative analysis

**Figure 5.**
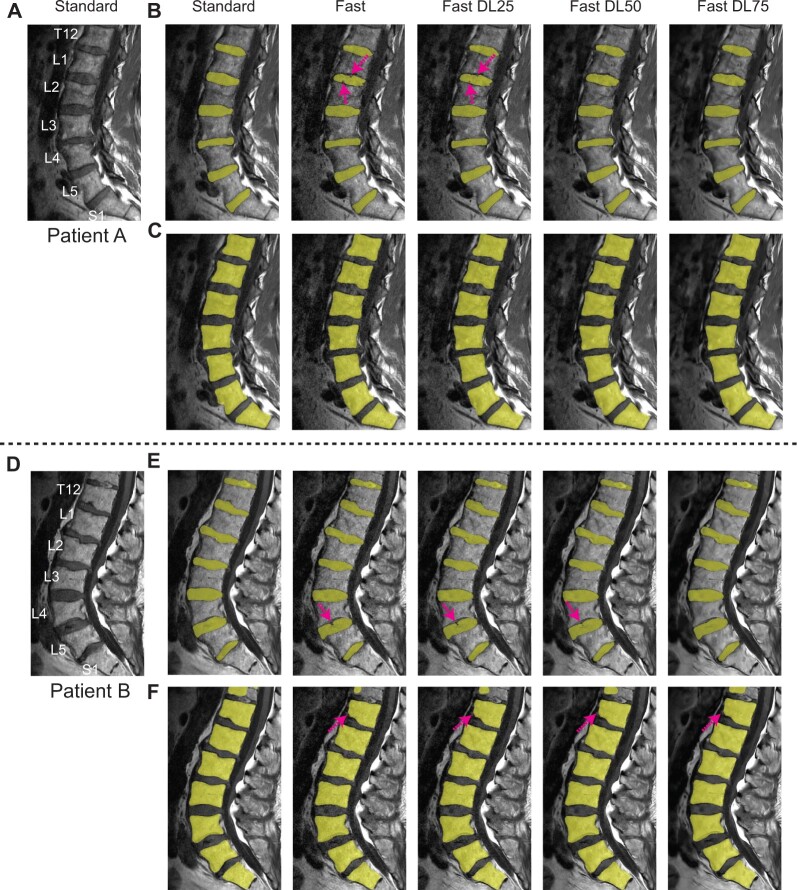
Disc and vertebral body segmentation masks. (**A, D**) Standard SAG T1 images, and segmentation masks of discs (**B, E**) and vertebral bodies (**C, F**) attained from Standard, Fast, and Fast-DL images of two patients. Some pixels within the disc regions were missed on Fast images, as denoted by arrows in (**B, E**), but disc segmentation was improved on Fast-DL images. For vertebral bodies, segmentation worked well over all the vertebral bodies except the T12 vertebral body on Fast and Fast-DL images in Patient B (**F**).

Figure 5 shows standard SAG T1 images (A, D) and compares segmentation masks of intervertebral discs (B, E) and vertebral bodies (C, F) attained from Standard, Fast, and Fast-DL images of two patients. Patient A was the same with the patient in Figure 1. Segmentation masks shown were right after applying convolutional neural networks and before applying additional post-processing. With Standard images, all discs were segmented well; however, with Fast images and Fast-DL images using a lower noise reduction factor, some pixels within the disc regions were not included for the L1–L2 disc mask (Patient A) and L4–L5 disc mask (Patient B), respectively. By incorporating DL reconstruction with a 50% or 75% noise reduction factor, both discs were segmented well, similarly to Standard images. For the vertebral bodies, segmentation worked well with Fast images and the Fast-DL images except the T12 vertebral body that included some imaging artifacts at the interface of the vertebral body and fat, probably caused by 0.5 NEX (partial Fourier) acquisition.

**Figure 6.**
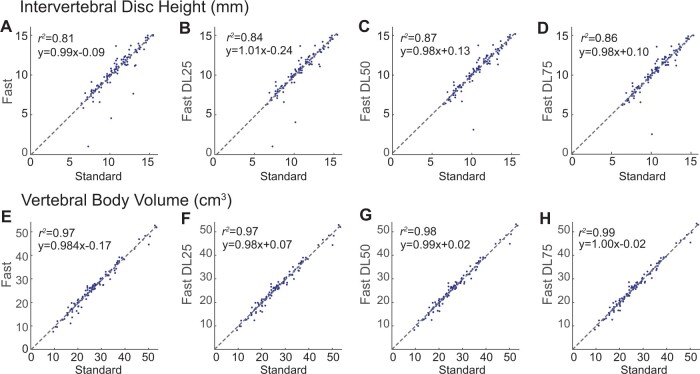
Correlation plots over extracted biomarkers. Disc heights (**A–D**) and vertebral body volumes (**E–H**) measured from Fast and Fast-DL images are compared to those from Standard images. Linear equations and the correlation of determination (*r*^2^) are denoted. The highest correlation was achieved when the noise reduction factor was 50% for disc heights (*r*^2^ *=* 0.87) and when the noise reduction factor was 75% for vertebral body volumes (*r*^2^ = 0.99).

Correlation plots of intervertebral disc heights and vertebral body volumes extracted from segmentation masks of Fast and Fast-DL images vs those from Standard images are shown in Figure 6. Linear equations and the correlation of determination (*r*^2^) were also denoted. Significant correlations were observed for all cases for both the intervertebral disc height and vertebral body volume. By applying DL reconstruction, *r*^2^ further increased, and the highest *r*^2^ was achieved when the noise reduction factor was 50% for disc heights (*r*^2^ = 0.87) and when the noise reduction factor was 75% for vertebral body volumes (*r*^2^ = 0.99). Table 6 compares the mean and standard deviations of extracted disc heights and intervertebral body volumes over the segmented discs and vertebral bodies. The mean and standard deviation (SD) of disc height from Standard images was 10.69 ± 2.30 mm, and the difference was −1.31%, −0.84%, and −0.84% for Fast, Fast DL25, Fast DL50, and Fast DL75 images, respectively. The mean intervertebral body volume of Standard images was 26.98 ± 9.33 cm^3^, and the difference was −2.19%, −1.45%, −0.79%, and −0.52% for the other image groups, respectively.

**Table 6.** Measured disc heights and vertebral body volumes based on automatic DL segmentations for each image group, and coefficients of determination between Fast/Fast-DL images and Standard images

	Intervertebral Disc Height		Vertebral Body Volume	
Images	Mean ± SD (mm)	Coefficient of Determination (*r*^2^)	Mean ± SD (cm^3^)	Coefficient of Determination (*r*^2^)
Standard	10.69 ± 2.30	-	26.98 ± 9.33	-
Fast	10.50 ± 2.52	0.81	26.39 ± 9.30	0.97
Fast DL25	10.55 ± 2.52	0.84	26.59 ± 9.28	0.97
Fast DL50	10.60 ± 2.41	0.87	26.74 ± 9.34	0.98
Fast DL75	10.60 ± 2.43	0.86	26.84 ± 9.38	0.99

## Discussion

In this work, we demonstrate that fast image acquisition with DL reconstruction has a potential to provide clinically diagnostic image quality for lumbar spine MRI while significantly shortening the exam duration. Additionally, we showed that convolutional neural networks trained with standard clinical images can reliably segment both intervertebral discs and vertebral bodies on Fast-DL images. Our patients had various LBP-associated imaging findings including degeneration in discs, vertebral bodies, facet joints with spinal canal and foraminal nerve compromise, while some patients had prior surgery, thus increasing the generalizability of our assessment and analysis to the broad-spectrum of clinical pathologies.

Although the radiologists evaluated “Apparent SNR” using a 1–5 scale without any quantitative measurement, we found that their mean “Apparent SNR” scores were, to some extent, in agreement with the expected SNR variations across different imaging groups. Fast images had a theoretical SNR reduction of 29/50/29% compared to Standard images, based on NEX differences, for SAG T2 FS, SAG T1, and AX T2, respectively. The mean scores for Fast images were lower than those for Standard images for all sequences. With SAG T2 FS and AX T2, DL reconstruction with noise reduction factors of 25% or higher provided mean scores that were noninferior to those of Standard images, while with SAG T1, only DL reconstruction with only a noise reduction factor of 75% provided a noninferior score (the *P* values for Fast DL50 images of SAG T1 for non-inferiority was .079).

For comparing qualitative scores between different imaging groups, we used a noninferiority test^38^^,^^39^ instead of a standard significance test. A significance test is to determine whether there is a significance difference between groups (ie, one group is superior to the other) by checking if the *P* values is smaller than .05. However, with this test, justifying similarity with *P* ≥ 0.05 is not appropriate as a high *P* values can be resulted from a lack of evidence in difference or a lack of statistical power due to a small sample size.^45^ An equivalent/noninferiority test has been specifically proposed to demonstrate similarity by checking whether the confidence interval of the difference is within a predetermined equivalence or noninferiority margin. In this study, we used the noninferiority test as we wanted to prove whether Fast-DL images are not worse than Standard images (can be equivalent or superior) rather than these are equivalent to Standard images. There is no standard about what should be noninferiority/equivalent margins for radiology research yet,^46^ but here we used the noninferiority margins of −0.5 for a 5-point scale and 0.2 for a binary scale, which were 12.5 and 20% of total ranges of scores respectively.

Based on qualitative assessment, the Fast-DL images with all of 25, 50, and 75% noise reduction factors from SAG T2 FS were noninferior (comparable or superior) to the standard clinical images in all categories, “Apparent SNR,” “Ability to Discern Anatomical Structures,” “Diagnostic Confidence,” “Overall Image Quality,” and “Presence of Artifacts.” With either SAG T1 or AX T2, Fast DL50 and Fast DL75 images provided statistically noninferior scores for four out of 10 comparisons. However, statistically inferior scores were recorded for “Overall Image Quality” and “Presence of Artifacts” on Fast DL50 and Fast DL75 images in both sequences. We believe that this was not due to problems with DL reconstruction, but was rather due to enhanced motion/flow artifacts with partial Fourier acquisition (0.5 NEX) of the fast protocol, and DL reconstruction might make the artifacts more apparent due to increased SNR. Enhanced wrapping/ghost artifacts with DL reconstruction have been previously reported.^27^^,^^29^ For the fast protocol, parallel imaging can be also used by exploiting multi-coil elements of posterior array coils. We expect motion/flow artifacts would not be that enhanced with the use of parallel imaging. For reconstruction, parallel imaging reconstruction can be applied to fill missing *k*-space data first, followed by DL reconstruction.

There was interobserver variability when assessing image quality by three radiologists. We observed that radiologist 1 (neuroradiologist) perceived standard images to have overall better image quality, while radiologists 2 (MSK radiologist) and 3 (neuroradiologist) were favorable to DL-reconstructed images, yielding the lowest Conger’s coefficients for Fast DL75 images. These variabilities made demonstrating statistical noninferiority more challenging. Radiologists have inherent biases, with some preferring realistic image textures and others sharper and smooth images,^47^ similarly to what DL reconstructions provide. In addition, differences in clinical disease experience between neuroradiologists and MSK radiologists may further explain the variability in interpretation of spine imaging with differing anatomical focuses.^48^ To exploit DL-reconstructed images in clinics, there is a need to validate DL-reconstructed images with many more radiologists with different background.

Despite the presence of interobserver variability, DL-based segmentation algorithms that were trained using clinical standard SAG T1 images worked well for Fast-DL images. Segmentation results visually looked better on Fast-DL images than on Fast images, and extracted disc height and vertebral body volumes were better correlated between Fast DL50/Fast DL75 images and Standard images. Even though further investigations are needed, the DL-reconstructed images seemed to be robust to machine algorithms.

This study has several limitations. First, the sample size of this study might not be sufficient to fully evaluate the ability of DL reconstruction in a wide range of lumbar spine pathologies. Secondly, the methods for qualitative assessment by radiologists can be improved. For example, the “Ability to Discern Anatomical Structures” was rated as one score, even though there are multiple complicated structures to evaluate. Individual scorings for delineation of vertebral bodies, discs, spinal cords, nerve roots, and facet joints, as used in studies such as Bratke G et al.^49^ or Sun et al.^50^ can allow for more comprehensive and objective evaluation. Furthermore, the presence of artifacts was rated as a binary score, but it may not be appropriate to evaluate the overall level of artifacts from various sources. Additionally, the ratio of NEX used between the fast and standard protocols was not equivalent between difference sequences. If the ratio had been kept equivalent, the effects of DL reconstruction might have been fairly evaluated between different sequences. Another issue might have arisen from MR data acquisition order during patient scans; fast protocol acquisitions were always performed after standard protocol acquisitions. This might have resulted in more motion artifacts in Fast images though we believe the effects would be minimal as imaging time for the fast protocols were under 10 minutes. Randomization of the order might have eliminated this potential problem. Lastly, these fast scans were not directly compared with other known fast imaging methods such as partial Fourier, parallel imaging, and compressed sensing, and data from only one vendor’s MRI scanner were involved in this study.

## Conclusions

This study demonstrates that DL reconstruction combined with fast acquisitions has a potential to provide diagnostic image quality noninferior to standard-care-of images. The difference in qualitative scores between Standard images and Fast-DL images varied with image parameters and sequences, but overall, this lumbar spine protocol, which was 52% faster, was able to provide scores noninferior to the standard protocol for apparent SNR, visualization of anatomical structures, and diagnostic confidence. However, larger studies involving multiple vendors are needed to assess the accuracy of these fast scans in diagnosing various pathologies and to compare the performance with other fast imaging methods such as parallel imaging and compressed sensing. We also validated that previously trained convolutional neural networks based on standard clinical SAG T1 images can reliably segment discs and vertebral bodies of DL-reconstructed images. The combination of fast acquisition, DL reconstruction, and DL-based image analysis will potentially enable a path for an efficient clinical workflow for patients with LBP.
